# New microbial tools to boost restoration and soil organic matter

**DOI:** 10.1111/1751-7915.14325

**Published:** 2023-08-08

**Authors:** Tadeo Sáez‐Sandino, Manuel Delgado‐Baquerizo, Eleonora Egidi, Brajesh K. Singh

**Affiliations:** ^1^ Departamento de Sistemas Físicos, Químicos y Naturales Universidad Pablo de Olavide Sevilla Spain; ^2^ Laboratorio de Biodiversidad y Funcionamiento Ecosistémico Instituto de Recursos Naturales y Agrobiología de Sevilla (IRNAS), CSIC Sevilla Spain; ^3^ Hawkesbury Institute for the Environment Western Sydney University Penrith New South Wales Australia; ^4^ Global Centre for Land‐Based Innovation Western Sydney University Penrith South DC New South Wales Australia

## Abstract

Anthropogenic activities are causing unprecedented rates of soil and ecosystem degradation, and the current restoration practices take decades and are prone to high rates of failure. Here we propose, the development and application of emerging microbiome tools that can potentially improve the contents and diversity of soil organic matters, enhancing the efficacy and consistency of restoration outcomes.

## Graphical abstract

Microbial restoration tools can simultaneously promote stocks and diversity of SOM and restoration outcomes.

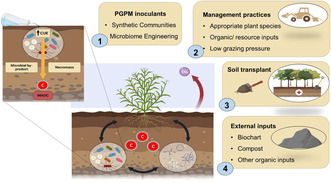

## INTRODUCTION

**FIGURE 1:**
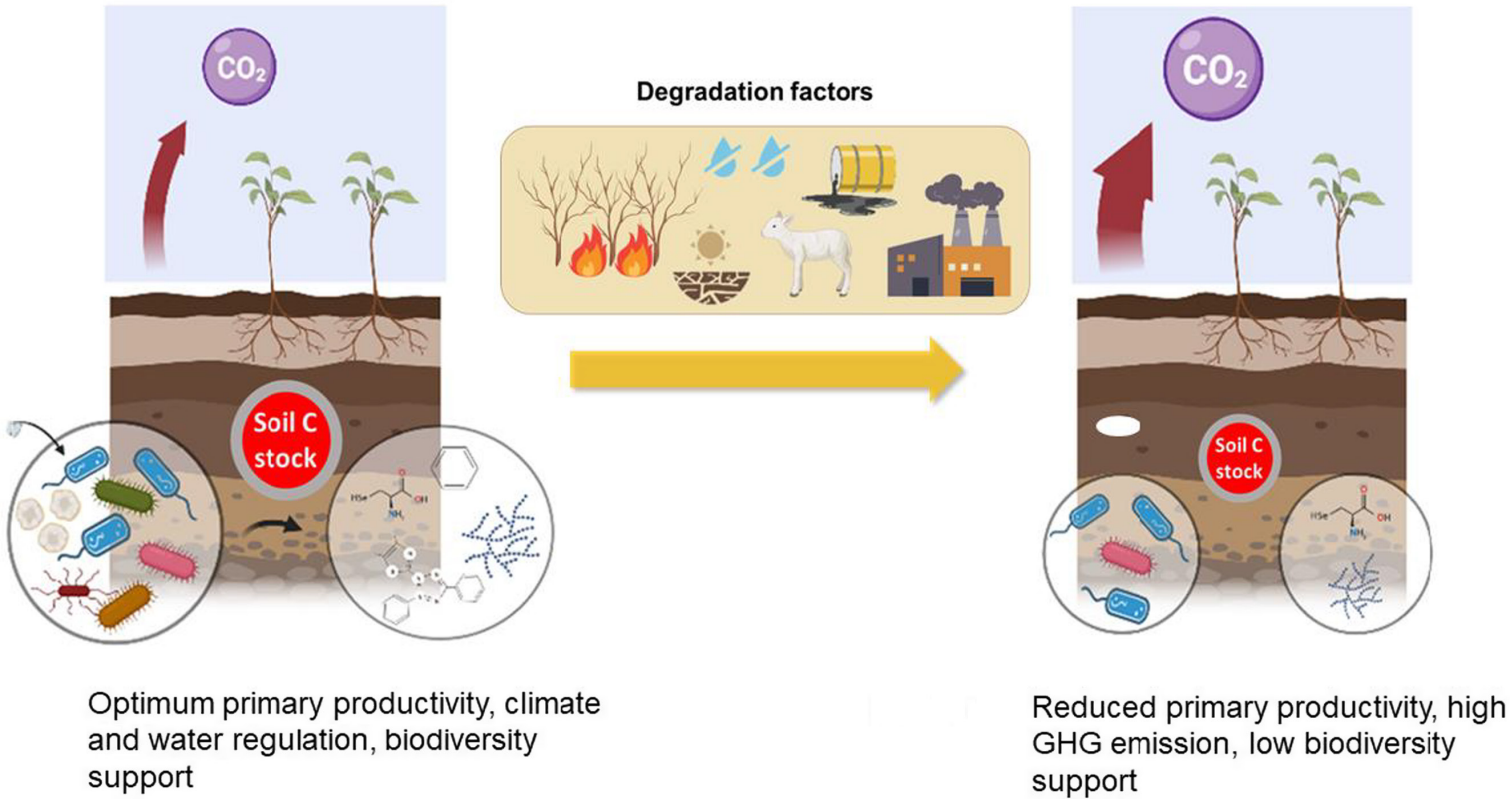
Drivers and impacts of soil degradation. Healthy soil supports multiple ecosystem functions including optimum primary productivity, climate (low greenhouse gas emission) and water regulations and habitats for biodiversity. However, soils are being degraded at an unprecedented rate caused by a multitude of anthropogenic factors including climatic change, extreme weather events and inappropriate management practices (e.g. land use, bushfires, contamination, deforestation, overgrazing). These factor lead to a reduction in soil organic matter stocks, high GHG emissions, and the loss of biodiversity and primary productivity.

The combination of climate change, population growth and land degradation (i.e. reduction or loss of the biological or economic productivity of the land) results in the loss of 24 billion metric tons of fertile soil per year (Coban et al., 2022). Moreover, soil‐degrading processes (e.g. drought, erosion, inappropriate land management practices and landslides) contribute to critical losses in soil organic matter (SOM) with negative consequences for soil biodiversity, fertility and primary productivity (Figure 1). Given that one‐third of all land areas in the world are already degraded to some extent (Cherlet et al., 2018), losses in soil fertility are expected to cause the displacement of approximately 50 million people over the next decade. Yet, the successful implementation of restoration strategies offers a sustainable solution with multiple societal co‐benefits, which explains its expansion during the UN Decade on Ecosystem Restoration (2021–2030). For instance, an approach based on land restoration could boost global agriculture productivity by 2%, contribute towards the reduction of biodiversity loss by 11% and increase ecosystem carbon (C) storage by 17 GtC by 2050 (Berkers, 2021; Mason et al., 2023). Thus, despite the restoration of natural and managed ecosystem functioning may take decades and is subjected to failure given the complexity of biological systems, it is vital that we identify strategies to boost restoration rates. For this purpose, multiple technologies have been proposed for the effective restoration of degraded land, including soil microbiome‐based tools, which are considered one of the most promising long‐term solutions. In this article, we explore the potential and challenges of microbial biotechnology tools to restore SOM stocks and diversity, which will favour production systems at the earliest possible timeframe and healthy soils with multiple co‐benefits (food supply, income for smallholder farmers, climate change mitigation, improved human and planetary health) directly relevant to the United Nations Sustainable Development Goals.

## OPPORTUNITIES FOR SOIL MICROBES TO BOOST SOM STOCKS IN DEGRADED SOILS

A critical point in soil restoration is to promote SOM accumulation, and thus, its multiple associated ecosystem services including soil fertility, biodiversity, C sequestration, water quality, resistance to erosion and primary productivity. The main objective of the SOM stocks is to support rapid and self‐sustained restoration favouring circular feedback of increased primary productivity. For instance, the SOM stocks promote soil biodiversity (i.e. nutrient cycling that enhances plant available nutrients), soil fertility and water storage, which provides ideal conditions for plant growth. Higher primary productivity means that plants are able to invest more carbon in soil via litter and rhizodeposition, further increasing SOM stocks. Thus, restoration tools that increase SOM stocks on degraded lands could help re‐establish the positive feedback cycles between soil biodiversity, fertility and plant productivity implied in natural ecosystems (Delgado‐Baquerizo et al., 2017).

The importance of soil microbes in processing SOM compounds is widely accepted (i.e. soil C losses from the soil due to decomposition of biomass by microbial respiration), but the role of the soil microbiome as a primary pathway of SOM formation (i.e. the C retained in soils by the formation of stable organic matter) has historically been overlooked (Kallenbach et al., 2016). Concretely, a myriad of microbially‐mediated mechanisms can promote SOM formation and storage. First, the SOM is mainly comprised of a diverse array of reactive C molecules, from plant inputs via litter (stems, leaves) and rhizo‐ (roots and exudates) depositions to microbially derived compounds (e.g. chitin). This interplay causes the composition of SOM to be classified into labile forms (e.g. carbohydrates that are the main source of C assimilated by soil biodiversity) and recalcitrant low‐quality substrates (e.g. aromatics produced by microbial activity). For example, a group of microbes (e.g. Actinobacteria) directly fix atmospheric CO_2_ into recalcitrant C via Wood‐Ljungdahl and Oxalate‐carbonate pathways (Mason et al., 2023). Second, the mineral protection of SOM is also broadly divided into particulate organic matter (POM; predominantly of plant origins) and mineral‐associated organic matter (MAOM; largely made of microbial products), which is more stable and less sensitive to climatic changes than the former (Cotrufo et al., 2019). Together, it is well documented that recalcitrant C forms of fine fractions (<0.4 mm) are stabilized in the MAOC fraction (Kirkby et al., 2014), which improves soil structure (e.g. aggregate stability, porosity) and soil capacity to hold plant available nutrients and waters that support higher plant productivity (e.g. see Li et al., 2023 where 77%–82% of fungal CO_2_ fixation was partitioned into the MAOC fraction). Thus, soil microbiomes play a fundamental role in controlling SOM formation, types and storage, indicating that a fast and successful restoration of terrestrial ecosystems could be achieved by manipulating the soil microbiome. These investigations highlight that the microbial approach could be considered as a game‐changer in the restoration of degraded lands (Wubs et al., 2016).

Moreover, SOM stocks also contain significant amounts of macronutrients (e.g. nitrogen, phosphorus, potassium and sulfur), which are released in plant‐available form through mineralization and solubilization processes carried out by soil microbes. In arid and hyper‐arid deserts, where the growth of plant primary producers is difficult due to lack of water and soil structure, cyanobacterial photosynthesis plays a dominant role in C fixation (see Maestre et al., 2017). In other environments, bacteria communities could increase photosynthesis rates through an enhanced supply of nutrients (e.g. N_2_ fixing and P solubilizing bacteria increase the availability of N and P for plant growth), whereas arbuscular mycorrhizal fungi could also enhance plant P uptake (Zhang et al., 2023). Similarly, microbes with higher C use efficiency (CUE) and slow‐growing microbes are expected to contribute to the development of more stable C stocks by reducing the portion that is pumped back into the atmosphere. A previous study reported up to a 17% increase in soil C over 14 weeks mediated by several melanized endophytic fungi (Mukasa Mugerwa & McGee, 2017). In addition to the provision of key nutrients, plant growth‐promoting microbes (PGPM) provide phytohormones and chelate metals to make them available to plant roots (Batista & Singh, 2021), while also providing biological control for protecting plants from pests, parasites or diseases (Backer et al., 2018). Together, soil microbes (from cyanobacteria to fungal mycorrhizal) are especially important in helping to build plant C storage, which could be an important first step to induce SOM formation and storage in highly degraded ecosystems.

**FIGURE 2:**
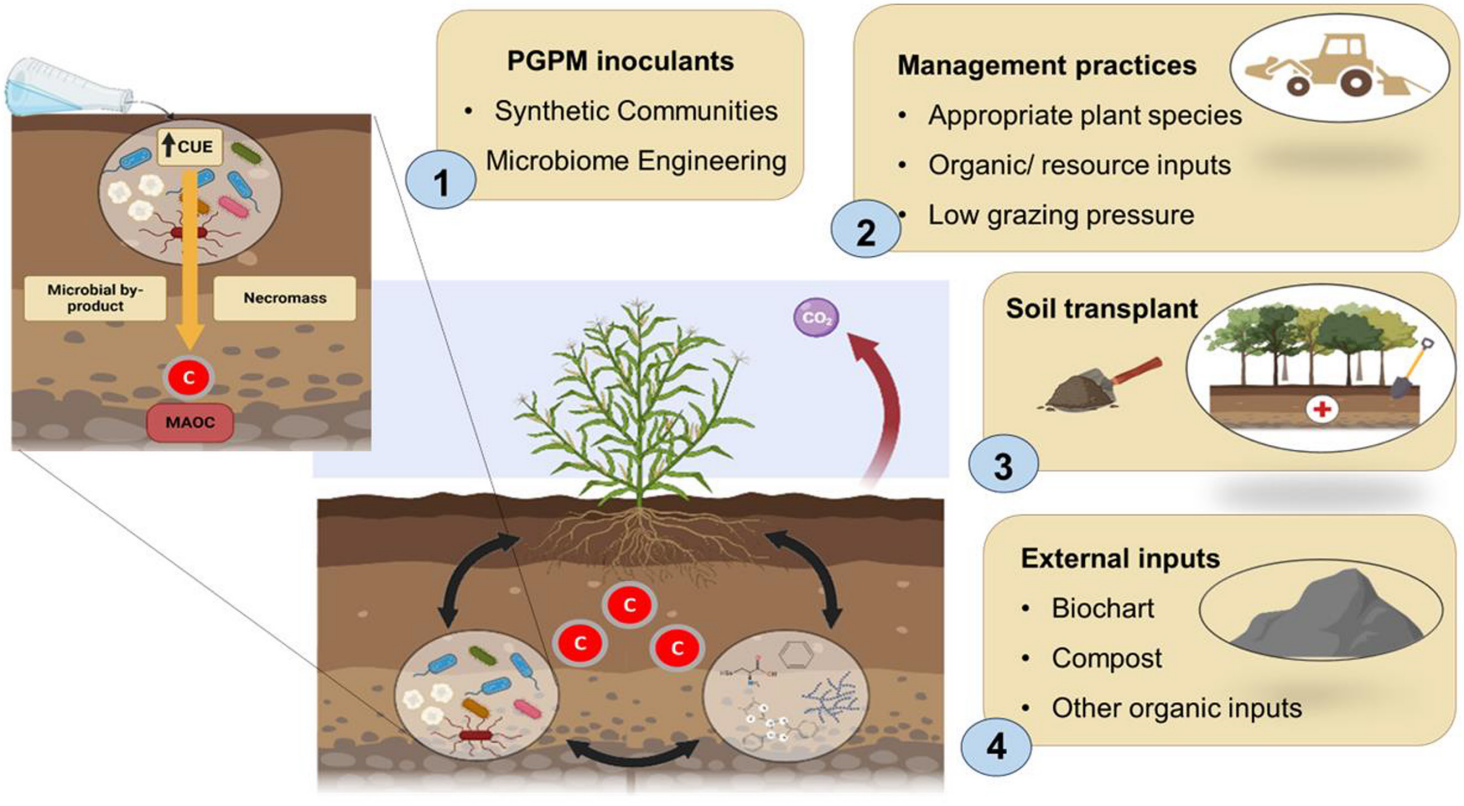
Microbial restoration tools that also promote soil organic matter (SOM) stocks and diversity (created with BioRender.com). The microbiome can be harnessed to restore SOM stocks and biodiversity: This includes: (1) Inoculation with plant growth‐promoting microbes PGPM, or synthetic communities (*SynComs*) or/and use of emerging tools of in situ microbiome engineering, (2) use of improved management practices that includes the use of appropriate plant species and organic inputs, and optimum grazing, (3) application of emerging tools such as soil transplant from healthy ecosystems that brings biodiversity and improve soil structure and (4) *the use of external inputs such as biochar or compost can enhance biological activities and can also improve soil structure over time*. Further microbial tools can be targeted on promoting microorganisms that favour by‐products and necromass over cellular respiration (e.g. higher microbial CUE), enhance microbial diversity with different metabolic pathways or use an approach that encompasses both strategies. The role of SOM molecular diversity on residence time could favour carbon increases over time. Combined strategies of inputs (e.g. PGMs microorganisms; organic resource, plant seeds) and improvement (e.g. soil aggregation via bacterial secretion and fungal hyphal extension) in conjunction with current practices (e.g. using vegetation) likely enhance the success of restoration strategies.

Overall, the manipulation of environmental microbiomes, along with the plant community, is emerging as a potentially effective intervention to increase SOM content (Albright et al., 2022). Yet, the microbiome approach fails frequently fails to establish long‐lasting modifications to ecosystem function because of the efficacy of microbial products (Timmis et al., 2017). Conversely, planting vascular plants makes worse outcomes in some ecosystems when they are introduced without considering ecological context (e.g. tree restoration can diminish water resources or reduce biodiversity; Maestre & Cortina, 2004). Even so, current restoration approaches primarily focus on the use of plants rather than microbes (Yang et al., 2022). Some practitioners and studies also support the use of compost, biochar or plants in combination with PGPM, but restoration outcomes from such approaches have been inconsistent. The key constraints of the restoration outcomes can be caused by the complex interplay between climatic and abiotic conditions, the ability of plants and microbes to colonize degraded poor‐nutrient land, the establishment of an improved microbiome in a recipient system of interest, and shifts in the relationship between plant and microbes in harsher conditions. We argue that a more holistic approach, integrating emerging tools with traditional methods, can provide improved solutions (Figure 2). Below, we outline two such approaches: harnessing SOM molecular diversity and integrating emerging microbial tools.

## EMBRACING A NEW PARADIGM FOR SOM MOLECULAR DIVERSITY IN RESTORATION STRATEGIES

The molecular diversity of SOM is complex and has emerged as a potentially critical control on the residence time of soil organic carbon (SOC). Current understanding suggests that higher SOM diversity requires higher microbial investment to produce enzymes, which causes slow microbial decomposition and leads to greater SOM accrual (Lehmann et al., 2020). Although empirical evidence has not yet been provided, we propose that promoting SOM diversity in the early stages could enhance the outcome of restoration activities. For example, soil degradation is accompanied by reduced microbial activity, diversity and plant C inputs. Even the accumulation of root necromass could stimulate microbial oxidative enzymes degrading recalcitrant organic matter, which would further reduce C storage (Breidenbach et al., 2022). Thus, it is plausible that the initial phase of soil restoration is characterized by a drastic reduction in molecular diversity coupled with a faster C mineralization.

Soil microbes can have a twofold impact on molecular diversity: by breaking down plant tissue into low‐molecular‐weight molecules, and by releasing microbial metabolic by‐products (e.g. primary and secondary metabolites, cell wall fragments). In fact, emerging evidence suggests that microbial CUE is at least four times as important as other factors (e.g. carbon inputs or decomposition) in determining SOC storage across contrasting global biomes (Tao et al., 2023). This result is supported by a positive relationship between CUE and SOC, which indicates that the accumulation of microbial by‐products and necromass favours SOC storage. Moreover, a more diverse community likely harbors more metabolic pathways for the consumption of the most common molecules, and they would generate microbial by‐products that are dissimilarly oxidized throughout the soil profile. In this regard, the use of certain types of soil microbes with C allocation to biomass and by‐products (as opposed to cellular respiration), along with increased microbial diversity favouring diverse metabolic pathways, could promote SOM diversity. As a result, and as per recent ecological theories (Lehmann et al., 2020; Tao et al., 2023), it is likely that increasing SOM diversity may enhance C accumulation over time.

It is likely that approaches based on external C supply (e.g. management practices such as compost or biochar from diverse plant communities) may also theoretically promote SOM diversity. However, the low capacity to store SOM due to the lack of mineral aggregates and soil in degraded soils hinders its applicability. Further interventions may be required in this case, for example, by mixing small amounts of healthy soils with minerals and other products (e.g. polymers) that could provide protection and long storage for SOM in soils. Moreover, increased plant diversity could promote microbial diversity, and ultimately, the quantity and chemical diversity of residues with positive effects on SOM stocks and fertility (Tiemann et al., 2015). We propose that initial SOM intervention, followed by the promotion of diverse plant communities using pioneering or indigenous plant species which can grow at low nutrient and water concentrations, can facilitate early colonization of degraded land. This fast and successful colonization could sustain SOM diversity in the long term, and ultimately, expedite restoration rates.

## MICROBIAL TECHNOLOGIES THAT CAN CONTRIBUTE TO SOM DIVERSITY

The use of PGMPs microorganisms, which can be reintroduced as microbial inoculants, has been used both in agriculture and restoration practices but with limited success. Understanding the ability of microbial inoculants to colonize soils and plants in harsh environmental conditions is a first requirement to improve efficacy and consistency. Current inoculant products are not tested for their colonization efficacy, however, it was recently proposed that ecological frameworks, such as metacommunity and priority theories, can help to assess these essential characteristics of inoculants (Singh et al., 2023). The use of synthetic communities (*SynComs*) is increasingly proposed as a more effective approach because it can use multiple strains to provide complementary functions (e.g. N_2_ fixation, pathogen or water deficit resistance) or the same functions in multiple species ensuring functional redundancy (Delgado‐Baquerizo, 2022). However, this approach also faces similar knowledge gaps as single microbial inoculants and necessitates systematic studies to ascertain their ability to colonize new habitats and understand how it is influenced by soil properties and climatic conditions.

The manipulation of the microbiome in situ could play a significant role in restoration as it has the potential to promote plant productivity while steering systems towards higher productivity. For instance, some agricultural management practices (e.g. no‐tillage and low N supply), promote members of Acidobacteria, Actinobacteria and fungi, which are considered for their higher CUE. As such, these microbes can theoretically sustain SOM for longer because the labile C pool is retained in the microbial pool. Similarly, different types of external inputs (e.g. compost or biochar), along with the use of metabolites (chemoattractants derived from plant or microbes), plant cultivars or even the development of new plant cultivars (e.g. via gene editing), could provide feasible and directional shifts in soil microbiomes, but concerted research efforts are needed. Moreover, these approaches can be effectively utilized to enhance root systems and extend fungal mycelium, facilitating increased rhizodeposition in subsoils characterized by lower rates of microbial decomposition compared to topsoils. As a result, the SOM persistence within subsoil layers is extended, thereby promoting long‐term soil structure and soil health. An additional benefit is its impact on soil aggregation—that is, a key indicator of soil health enhancing soil structure, water retention and protecting soil C from degradation—via bacterial secretion and fungal hyphal extensions that may positively influence the rate of restoration. However, the current toolbox to manipulate the microbiome in situ has shown limited success under field conditions, mostly because of competition with indigenous microbes (Singh et al., 2023; Verstraete et al., 2021). Such limitation could potentially be overcome by activating dormant indigenous microbes using a microbial consortium (probiotics) in combination with stimulants (prebiotics), which could encourage microbial biomass and activity (Maestre et al., 2017).

Recent studies have demonstrated that the introduction of a small amount of soil transplant from healthy ecosystems can kick‐start a rapid rate of recovery and restoration (Afkhami, 2023). Yet, such an approach faces numerous challenges: (i) it can introduce pathogens, pests and invasive species, which can have long‐term impacts on ecosystem recovery; (ii) it could facilitate the spread of new species that displace indigenous microbes; (iii) the success rate is low (i.e. it requires a very large amount of healthy soil, which is unlikely due to logistical reasons). In this regard, the microbial communities found in healthy and degraded soils are expected to exhibit significant differences, leading to competition for niche occupation and known outcomes. However, recent advances in theoretical (e.g. microbial coalescence), experimental and modelling approaches can help guide a more effective solution. For instance, a stoichiometric‐based modelling approach with a focus on flux balance analysis (FBA) can help predict community colonization based on a metabolic network reconstructed for the whole community (Freilich et al., 2011). This is vital because microbial interactions present positive and negative associations that determine their abundance in soils (e.g. there is a competitive exclusion between members of *Bacillales* and *Proteobacteriales*; Romdhane et al., 2022). These microbiome tools, complemented by metagenomic‐derived information from environmental surveys, provide a golden opportunity to develop and employ effective restoration strategies.

Overall, there is an urgent need to develop and employ effective restoration approaches to address the multiple global challenges our society faces. Effective restoration can contribute to food security (by bringing degraded land into production systems), climate change mitigation (by increasing soil carbon stocks and plant storage), poverty alleviation (as a new source of income for smallholding farmers) and multiple ecosystem services on which humanity depends (ranging from clean water to pollution removal). However, available tools for restoration are limited and are mainly focused on using plants. Here, we propose an approach shifting the focus from a vegetation‐based solution to the inclusion of soil microbiomes and SOM for effective restoration tools. We believe explicit consideration of soil microbiomes and the SOM diversity can enhance both efficacy and consistency of restoration outcomes, given the central role of SOM in primary productivity through nutrient supply and soil health. Particularly, the use of microbial inoculants (e.g. *SynComs*), microbiome in situ manipulation, plant‐microbe products and soil transplant emerge as a significant promise, but our technical abilities to manipulate soil microbial communities remain in infancy. New analytical tools (meta‐omics, gene editing, CRISPR‐CAS) provide opportunities to systematically address those limitations. Once technical issues are resolved, combining soil, microbe and plant tools with artificial intelligence (e.g. choose the best inoculants or plant‐microbe combination) and satellite technologies (monitoring the performance of restoration approaches) can provide an effective solution for successful outcomes of restoration practices.

## AUTHOR CONTRIBUTIONS

**Brajesh K. Singh:** Conceptualization (equal); funding acquisition (equal); investigation (equal); supervision (equal); visualization (supporting); writing – original draft (supporting); writing – review and editing (equal). **Tadeo Sáez‐Sandino:** Conceptualization (lead); investigation (lead); methodology (equal); visualization (lead); writing – original draft (lead); writing – review and editing (equal). **Manuel Delgado‐Baquerizo:** Conceptualization (equal); funding acquisition (equal); investigation (equal); supervision (equal); writing – original draft (equal); writing – review and editing (supporting). **Eleonora Egidi:** Conceptualization (equal); funding acquisition (equal); investigation (equal); visualization (equal); writing – original draft (equal); writing – review and editing (supporting).

## FUNDING INFORMATION

BKS acknowledges funding from Australian Research Council (DP 210102081; DP230101448) on multitrophic interactions microbiome tools. EE is supported by an Australian Research Council fellowship (DE210101822). MD‐B is supported by the Spanish Ministry of Science and Innovation (PID202‐115813RA‐100). TS‐S is supported by the Spanish Ministry of Science and Innovation (CGL2017‐88‐124‐R).

## CONFLICT OF INTEREST STATEMENT

None.
